# Early Changes in Glutamate Metabolism and Perfusion in Basal Ganglia following Hypoxia-Ischemia in Neonatal Piglets: A Multi-Sequence 3.0T MR Study

**DOI:** 10.3389/fphys.2017.00237

**Published:** 2017-04-25

**Authors:** Yu-xue Dang, Kai-ning Shi, Xiao-ming Wang

**Affiliations:** ^1^Department of Radiology, Shengjing Hospital of China Medical UniversityShenyang, China; ^2^Department of Imaging Systems Clinical Science, Philips HealthcareBeijing, China

**Keywords:** hypoxic–ischemic brain damage, ^1^H-MRS, IVIM, glutamate, perfusion

## Abstract

The excitotoxicity of glutamate metabolism as well as hemodynamic disorders of the brain are both risk factors for neonatal hypoxic–ischemic brain damage (HIBD). In the present study, changes in glutamate metabolism in the basal ganglia were detected by proton magnetic resonance spectroscopy (^1^H-MRS) at 0–6, 8–12, 24–30, and 48–60 h after the induction of hypoxia-ischemia (HI) in newborn piglets. Meanwhile, correlation analysis was performed by combining the microcirculatory perfusion informations acquired by intravoxel incoherent motion (IVIM) scan to explore their possible interaction mechanism. The results suggested that Glu level in the basal ganglia underwent a “two-phase” change after HI; perfusion fraction *f*, an IVIM-derived perfusion parameter, was clearly decreased in the early stage after HI, then demonstrated a transient and slight recovery process, and thereafter continued to decrease. The changes in *f* and Glu level were in a significant negative correlation (*r* = −0.643, *P* = 0.001). Our study results revealed that Glu level is closely associated with the microcirculatory perfusion changes in the acute stage of HIBD.

## Introduction

Basal ganglia injury (BGI) is a common type of hypoxic–ischemic brain damage (HIBD) and also the main cause of permanent dysneuria and cerebral palsy in perinatal full-term neonates. The basal ganglia are very sensitive to hypoxia–ischemia (HI) damage and susceptible to selective neuronal injury (Martin et al., 1997; Rocha-Ferreira and Hristova, 2016). Glutamate (Glu), as the most important excitatory neurotransmitter in animals, plays a crucial role in maintaining the function of glutamatergic neurons in the basal ganglia (Alexander and Crutcher, 1990). The excitotoxicity caused by the massive accumulation of Glu after HI is the central link for HIBD and is also the initiator and executer of brain injury (Hagberg et al., 1987; Choi and Rothman, 1990; Coyle and Puttfarcken, 1993; Rego et al., 1996).

Normally the release and reuptake of Glu are in a dynamic balance. The efficient Glu uptake system enables low, extracellular concentrations of Glu in order to avoid excitotoxicity, and only a small amount of Glu is involved in signal transduction as an excitatory neurotransmitter (D'souza and Slater, 1995; Cooper and Jeitner, 2016; Danbolt et al., 2016). However, HI leads to an increase in Glu release and/or damages the Glu uptake system, thus causing a sharp elevation of extracellular Glu; its level and the excessive activation of relevant receptors contributes to nervous excitotoxicity and results in generalized pathological brain lesions. Of these excessively activated receptors, the N-methyl-D-aspartic acid receptor plays an important role in neuronal injury after mediating HI as outlined in our preliminary study (Wang X. Y. et al., 2012).

Perinatal HIBD has a complex pathophysiological mechanism. In addition to the excitotoxicity of Glu, hemodynamic disorders of the brain are also a risk factor for HIBD (Pryds et al., 1990; Howlett et al., 2013; Massaro et al., 2015). Hemodynamic disorders of the brain can contribute to secondary energy dysmetabolism after HI, and thus have a role in pathological mechanisms involved in brain damage secondary to HI. The pathogenesis of brain damage can be revealed by evaluating cerebral perfusion. For example, a pivotal question arises concerning cerebral metabolism in a HI environment in response to changes in cerebral perfusion: do mutual effects and interactions exist between such variables? In order to understand HIBD mechanisms, it is essential to explore the relationship between changes in Glu metabolism and changes in microcirculatory perfusion after HI.

Proton magnetic resonance spectroscopy (^1^H-MRS), as a non-invasive technique, provides information on changes of metabolites in brain tissues. At present, two major functional magnetic resonance imaging (MRI) techniques are available to measure cerebral perfusion: perfusion-weighted imaging (PWI) and arterial spin labeling (ASL). Considering the specificity of the newborns, invasive PWI cannot be used as it requires the use of exogenous contrast media. With regard to ASL, this can only evaluate a single parameter—cerebral blood flow (CBF)—which does not allow the all-round comprehension of a hemodynamic reserve. This leads us to the question of whether an MRI technique exists that can not only meet the special requirements of non-invasive examinations in newborns, but also support the comprehensive evaluation of cerebral perfusion? Intravoxel incoherent motion (IVIM), a non-invasive MR perfusion imaging technique, can quantify the microcirculation of blood in the capillary network (perfusion) and the diffusion composition of true water molecules in tissues *in vivo* using a bi-exponential model (Le Bihan et al., 1986, 1988). Measurements using IVIM are based on the premise that the microcirculation of blood, or perfusion, is a kind of non-uniform, irregular random motion (i.e., incoherent motion) (Le Bihan et al., 1988). A linear correlation exists between IVIM-derived perfusion parameters (pseudo-diffusion coefficient D^*^ and perfusion fraction *f*) and conventional perfusion parameters (Le Bihan and Turner, 1992; Federau et al., 2014a,b). In theory, therefore, IVIM-derived perfusion parameters can be used to evaluate the microcirculatory perfusion of the brain after HI and thus provide more comprehensive information. Currently, the successful application of IVIM in the central nervous system focuses on studies to recognize the ischemic semi-dark band (Federau et al., 2014b; Hu et al., 2015), identify the benignity and malignancy of tumors(Bisdas et al., 2014; Hu et al., 2014; Suh et al., 2014) and evaluate the efficacy of radiochemotherapy (Hauser et al., 2014; Cui et al., 2015; Xiao et al., 2015). In this context, IVIM, perhaps, will become a new MR perfusion technique useful in the study of perfusion of the microcirculation in newborns.

Studies are urgently required on the critical changes that occur at the Glu level during the development of HIBD and its relationship with microcirculatory perfusion. In this study, we measured Glu-related metabolites and microcirculatory perfusion separately by ^1^H-MRS and IVIM in a HI animal model. We investigated the dynamic changes in Glu and microcirculatory perfusion after HI and preliminarily explored their possible interaction mechanism.

## Materials and methods

### Preparation of experimental animals and establishment of HIBD model

All animal experiments were reported in accordance with Animal Research: Reporting of *In vivo* Experiments guidelines. This study was approved by the Institutional Animal Care and Use Committee of Shengjing Hospital of China Medical University. Twenty-five newborn male or female Yorkshire piglets (P3–5 d; weight: 1.5–2 kg) were randomly selected from the Laboratory Animal Center of Shengjing Hospital of China Medical University. All animal models were established according to the Regulations for the Animal Care and Use published by the Shengjing Hospital of China Medical University.

The newborn piglets were anesthetized by an intramuscular injection of 0.6 mL/kg Su-Mian-Xin (xylazine hydrochloride) in the buttocks. During anesthesia, the animals' vital signs were closely observed. When the piglets were found to become comatose, as evidenced by muscle relaxation, decreased limb muscular tension and a delayed corneal reflex, the animals were placed in a supine position on a bench prior to each operation. A laryngoscopic tracheal cannulation (φ 2.5 mm) was performed, and each animal was then connected to a TKR-200C small animal ventilator (Jiangxi Teli Anesthesia and Respiratory Equipment Co., Ltd, Jiangxi, China) for mechanical ventilation with 100% oxygen. Ventilator parameters were as follows: respiration ratio inspiration/expiration (I/E) = 1:1.5; and respiration frequency = 30 bpm. Heart rate and peripheral oxygen saturation were monitored using a TuffSat handheld pulse oximeter (GE, Boston, MA, USA). The incision area and adjacent skin were then disinfected and a median incision was made in the neck. Bilateral common carotid arteries and adjacent internal jugular veins and vagus nerves were dissected, and a 5.0 mm silk suture was indwelled. After the condition of animals stabilized for 30 min, the bilateral common carotid arteries were clipped with small artery clamps to interrupt their blood flow, and 6% oxygen–containing mixed gas was delivered mechanically for 40 min. Thereafter, the HI induction procedure was completed, the small artery clamps removed, and the blood flow of the bilateral common carotid arteries was recovered. Oxygen (100%) was mechanically delivered again, and the incision was sutured. After the operation, the animals were transferred to an incubator (37°C) to ensure a body temperature in the normal range during postoperative recovery. This well-established model was used to induce bilateral HI injury, as documented in our previous studies (Wang H. et al., 2012; Zhang et al., 2012).

### Grouping of experimental animals

All newborn piglets were randomly divided into the control group (sham-operation group, *n* = 5) and the HI model group (*n* = 20), and the HI model group was then further divided into 4 subgroups according to different time points after HI: 0–6, 8–12, 24–30, and 48–60 h (*n* = 5 per group). The animals in the pseudo-operation group underwent preoperative preparation similar to those in the model group but without a HI induction procedure.

### MR scans and data post-processing

At different time points after HI, a conventional MR scan was performed on all animals using a 3.0T MRI system (Achieva 3.0T TX; Philips Healthcare Systems, Best, The Netherlands) with an eight-channel phased array head coil. Images (including axial and sagittal T_1_-weighted images [T_1_WI] and axial T_2_-weighted images [T_2_WI] of the head) were acquired by fast gradient echo. The relevant scan parameters used were: T_1_WI Repetition time (TR) 200 ms; Echo time (TE) 2.3 ms; matrix 224 × 162; slice thickness 5 mm; T_2_WI TR 5000 ms; TE 80 ms; matrix 224 × 162; and slice thickness 5 mm.

**Figure 1:**
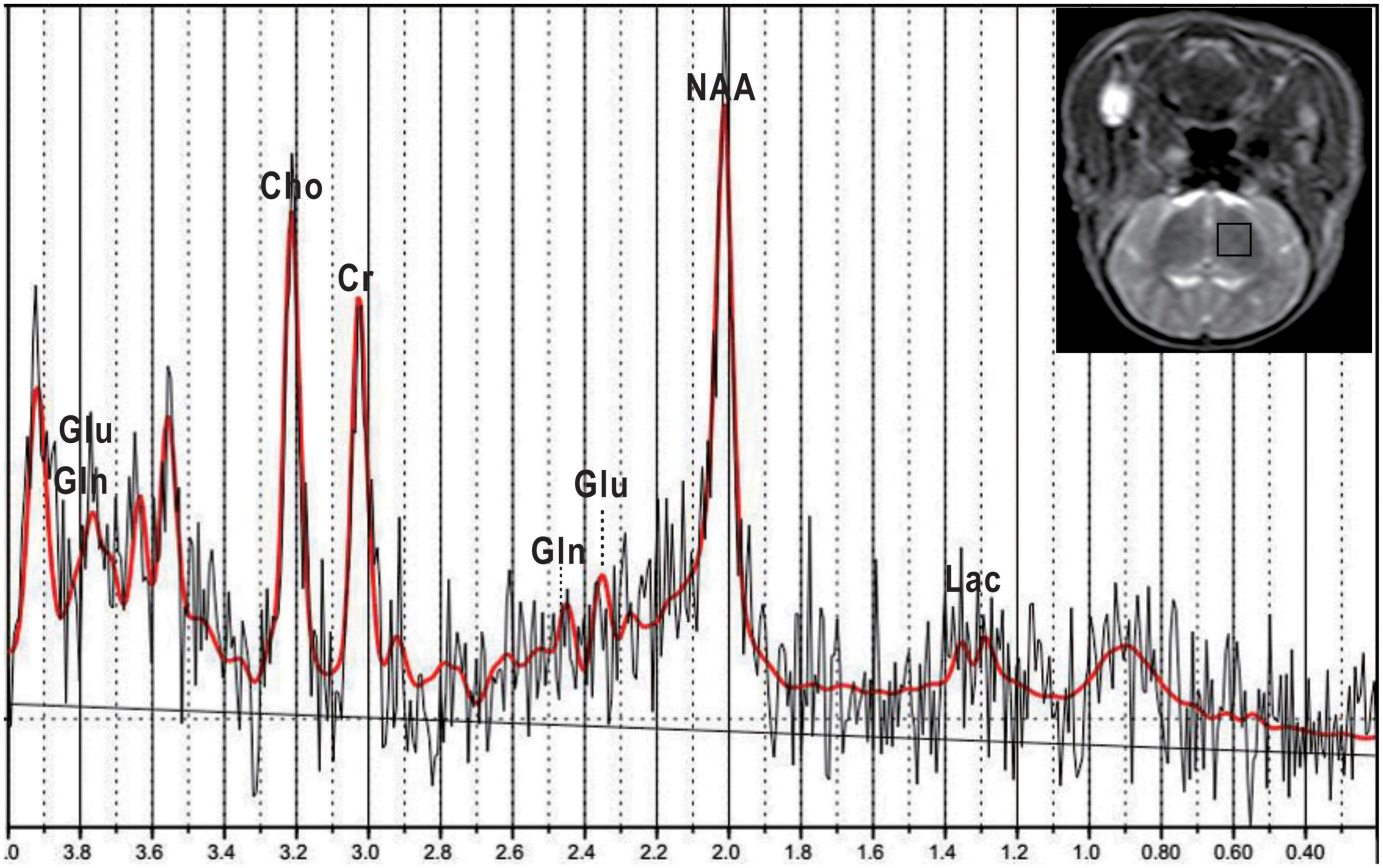
**Representative T_2_-weighted image and corresponding ^1^H-MRS of a normal piglet**. A MRS voxel was placed in the left basal ganglia (black square). LCModel fitting results (red), fit from 0.2 to 4.0 ppm, are presented. Glu, glutamate; Gln, glutamine; NAA, N-acetylaspartate; Lac, lactate; Cho, choline; Cr, creatine.

Measurements by single-voxel ^1^H-MRS were completed with a point-resolved spectroscopy (PRESS) sequence using one 90° and two 180° radio frequency pulses (13.2224 ms; bandwidth = 1,231 Hz). The following parameters were used: TR = 2,000 ms; TE = 37 ms; bandwidth = 2,000 Hz; number of signal acquisitions = 64; and a volume of interest (VOI; 10 × 10 × 10 mm) was positioned in the left basal ganglia. A plain scan was performed on newborn piglets to acquire coronal scan images of the basal ganglia as a region of interest (ROI). After the completion of a ^1^H-MRS scan, the raw data were then entered into LCModel (Linear Combination Model) software (version 6.3-1B) (Provencher, 2001) for quantification, with an unsuppressed water signal used as an internal reference, as well as automatic processing and analysis. The LCModel software supported automatic baseline corrections and the smoothing of spectrum raw data, as well as the absolute concentration measurement of several metabolites, including alanine (Ala), aspartic acid (Asp), creatine (Cr), phosphocreatine (PCr), γ-aminobutyric acid (GABA), glucose (Glc), glutamate (Glu), glutamine (Gln), glycerylphosphorylcholine (GPC), phosphatidylcholine (PCh), glutathione (GSH), inositol (Ins), lactic acid (Lac), N-acetylaspartate (NAA), N-acetylaspartate glutamate (NAAG), scyllo-inositol (Src) and taurine (Tau). Glu is the most abundant excitatory neurotransmitter in the brain and is essential for normal brain function; its complex signals were generated at 2.04–2.35 ppm (ppm: 10^−6^) and 3.75 ppm. The peak of Gln overlapped with that of Glu at approximately 2.35 ppm. LCModel software can automatically distinguish the overlapping signals of different metabolites with a similar chemical shift in the same frequency area (Wisnowski et al., 2013). The spectral fitting was performed with a range from 0.2 to 4.0 ppm (see Figure 1). In this study, we quantified the concentrations of Glu, Gln and Glx (Glu + Gln complex). Cramér–Rao lower bounds (CRLBs), which were provided by LCModel software, were used in assessing the precision of the quantification of metabolites. Spectrum data were included in the statistical analysis, which met the following criteria: (1) signal–to–noise ratio (SNR) ≥ 5; and (2) CRLBs for the concentrations of metabolites obtained were < 50%, and generally < 25%.

An IVIM scan was performed with a single-shot spin echo planar imaging (EPI) sequence; TR/TE 4,000 ms/90 ms; matrix 228 × 231; slice thickness 5 mm; and 16 *b*-values (0, 10, 20, 40, 80, 110, 140, 170, 200, 300, 400, 500, 600, 700, 800, and 900 s/mm^2^). IVIM analysis was performed using in-house MATLAB software (R2010a, The MathWorks, Inc., Natick, MA, USA) and a bi-exponential model. The relation between the signal attenuation and b is expressed by the following formula (Le Bihan et al., 1988, 1989):

(1) $$\begin{array}{r} {\text{S}}_{\text{b}}/{\text{S}}_{0}\;=\;\left(1-\text{f}\right)\cdot \text{exp}\left(-\text{b}\cdot \text{D}\right)+\text{f}\cdot \text{exp}\left(-\text{b}\cdot {\text{D}}^{*}\right) \end{array}$$

Where S_b_ and S_0_ stand for signal intensity at b ≠ 0 and b = 0, respectively. The relevant parameters finally obtained by least squares (Pfeuffer et al., 1999) are as follows: diffusion coefficient *D* (given in units of × 10^−3^ mm^2^/s) denotes the true molecular diffusion; the pseudo-diffusion coefficient D^*^ (given in units of × 10^−3^ mm^2^/s) represents the diffusion linked to microcapillary perfusion; and the perfusion fraction *f* (given as a percentage) indicates the proportion of microcirculatory perfusion-related diffusion in the total diffusion. As there is a variation in the speed of motion of different molecules, D^*^ is markedly greater than *D*. At *b* < 200 s/mm^2^, the perfusion effect is dominant and the signal attenuation detected reflects information from perfusion; at *b* > 200 s/mm^2^, the microcirculatory perfusion effect can be neglected, and the signal attenuation detected nearly reflects the true diffusion of water molecules in voxels.

**Figure 2:**
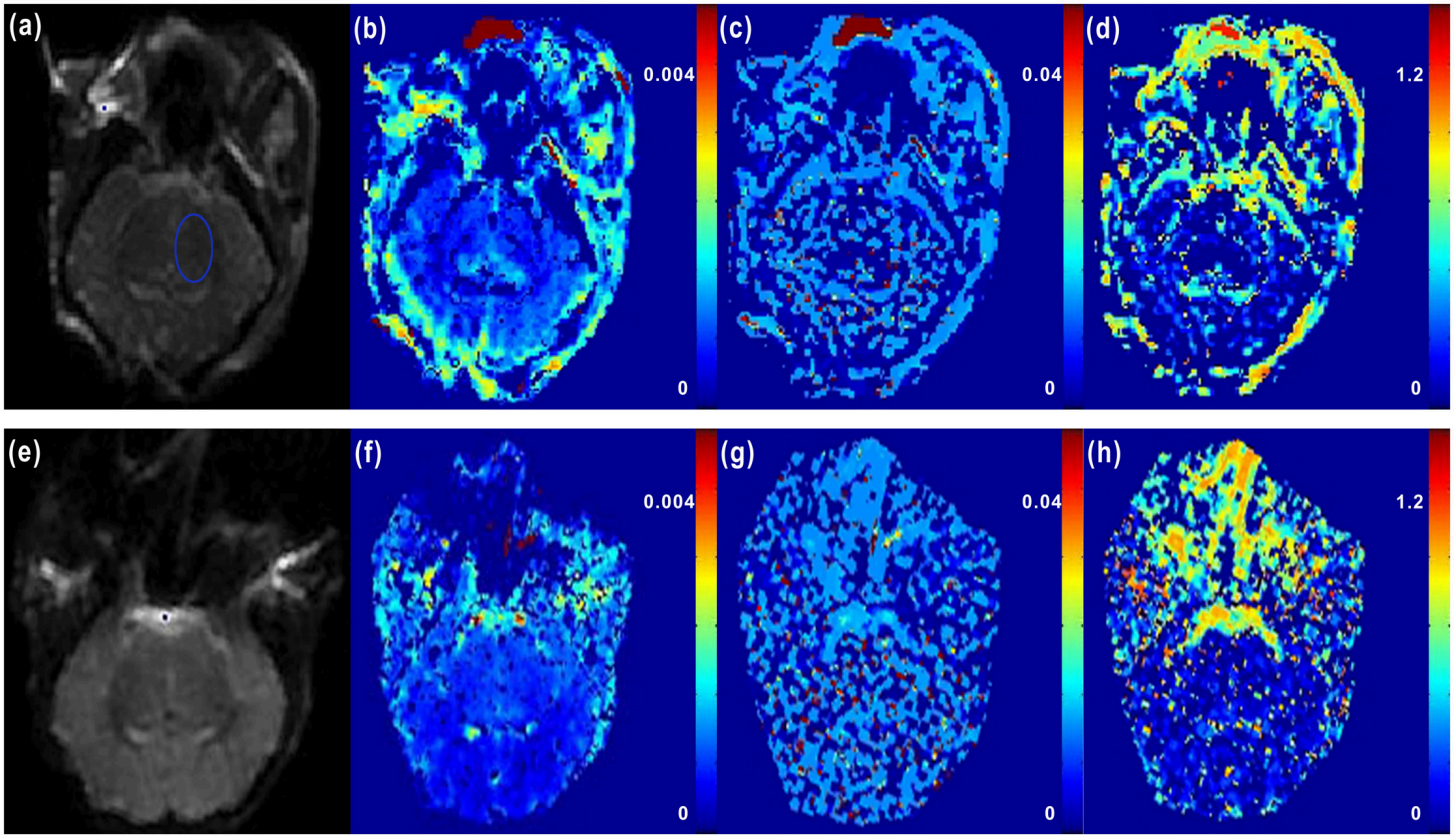
**Representative IVIM images of newborn piglets before and after HI**. **(a**–**d)** showed the axial diffusion-weighted imaging (DWI) image (*b* = 0 s/mm^2^) of a normal newborn piglet **(a)** (where the blue ellipse highlights the region of interest (ROI) marked in the left basal ganglia region), and the corresponding D, D^*^, and *f* images obtained by IVIM post-processing software (**b**–**d**; *D* = 0.642 × 10^−3^ mm^2^/s, D^*^ = 13.207 × 10^−3^ mm^2^/s, *f* = 12.753%). **(e–h)** showed the axial DWI image (*b* = 0 s/mm^2^) of a newborn piglet at 6 h after HI **(e)**, and the corresponding D, D^*^, and *f* images (**f**–**h**; *D* = 0.269 × 10^−3^ mm^2^/s, D^*^ = 5.412 × 10^−3^ mm^2^/s, *f* = 7.197%). From the axial DWI images, we could see that at 6 h after HI, the signals of cerebral parenchyma were evidently enhanced, the cerebral cortex was obviously swollen and edematous, and the cortical sulci and gyrus became shallower; in the *D* and *f* images, weakened signals of cerebral parenchyma were observed after HI correspondingly, but the difference in the image of D^*^ was not significant.

Firstly, IVIM raw data were imported into post-processing software, and then post-processed using a bi-exponential model to give pseudo-color images of D, D^*^, and *f*. Secondly, the ROI was manually marked on the left basal ganglia in images at b = 0 s/mm^2^ (see Figure 2) by combining T_2_WI scan images (avoiding the adjacent cerebrospinal fluid [CSF], blood vessels and noise areas), and then D, D^*^, and *f* values were calculated. If the calculated results were < 0, *f*, *D*, and D^*^ were set to 0 to comply with actual physiology. If *f* was > 0.3 and D^*^ was > 0.05 as calculated, *f* and D^*^ were also set to 0 because such calculated results may be caused by the effects of SNR or CSF flow, and were therefore not physiological values (Federau et al., 2012). Finally, the relevant parameters of ROI in model and control groups were measured repeatedly three times and results then averaged. Differences in various parameters between model and control groups were analyzed.

### Statistical analysis

SPSS 20.0 statistical software was used for analyses. All data were expressed as mean ± standard deviation (SD). Multiple comparisons of data with a homogeneity of variance were performed by one-way analysis of variance (ANOVA), and those of data with a heterogeneity of variance by a Kruskal–Wallis *H* test. Correlations between the Glu level and IVIM-derived perfusion parameters (D^*^ and *f*) were analyzed by Spearman rank correlation analysis. The correlation level indicated by the correlation coefficient was defined as follows: *r* > 0.8, very high correlation; 0.6 < *r* ≤ 0.8, significant correlation; 0.4 < *r* ≤ 0.6, ordinary correlation; 0.2 ≤ *r* ≤ 0.4, low correlation; and 0 ≤ *r* < 0.2, weak or no correlation (Fujima et al., 2014). *P* < 0.05 suggested that a difference was statistically significant.

## Results

In this study, ^1^H-MRS and IVIM scans were performed on the newborn piglets in the control group and HI model group at different time points of 0–6, 8–12, 24–30, and 48–60 h after HI. The results are described below.

### ^1^H-MRS

**Figure 3:**
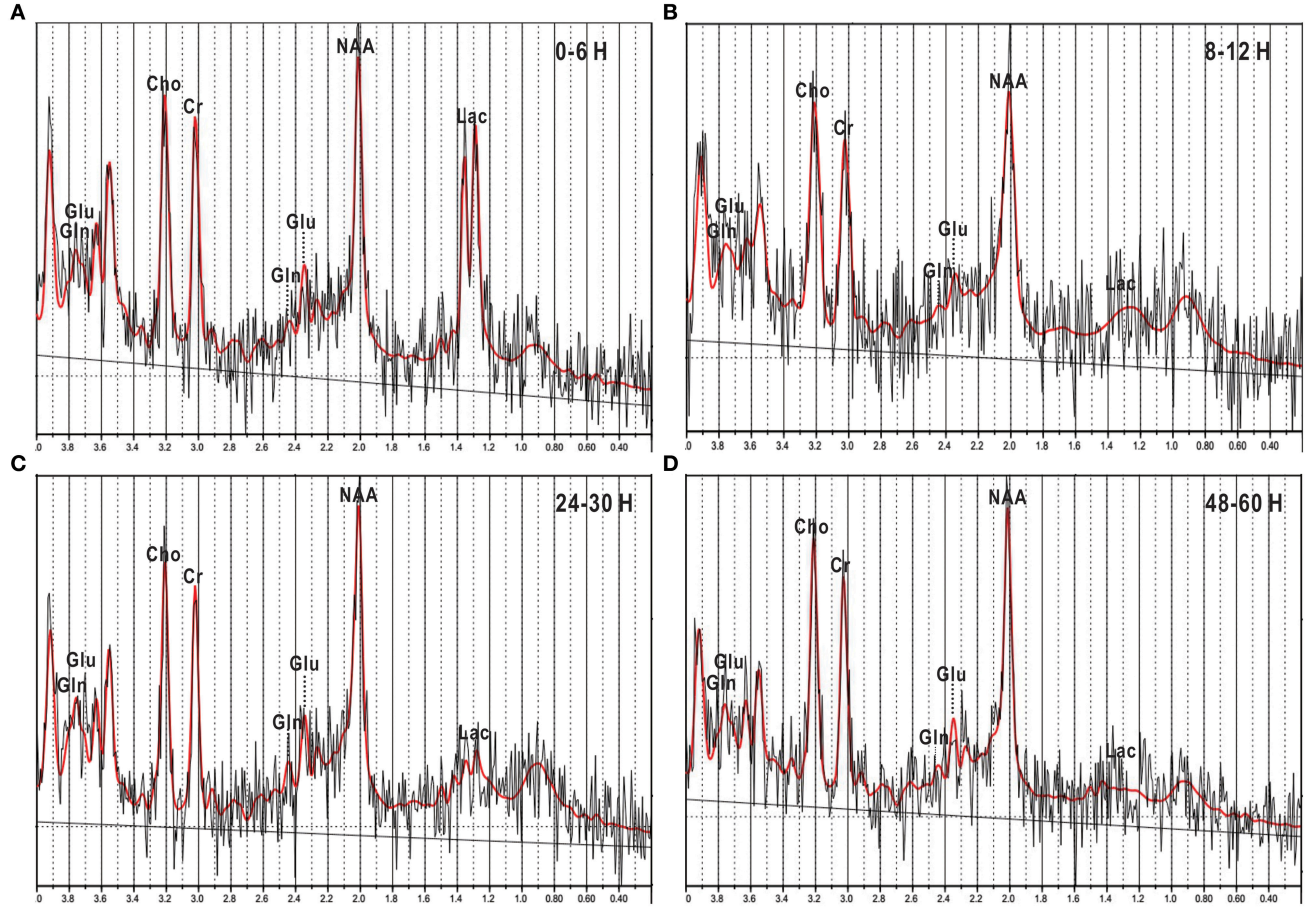
**^1^H-MRS images of newborn piglets at different times points after HI injury**. **(A–C)** and **(D)** represented four different time points after HI operation: 0–6, 8–12, 24–30, and 48–60 h. In the spectra, the Glu peak was elevated at 0–6 h **(A)**, then slightly depressed at 8–12 h **(B)**, and thereafter elevated again at 24–30 h **(C)**, and 48–60 h **(D)**. However, the Gln peak did not show significant changes in the above image. Glu, glutamate; Gln, glutamine; NAA, N-acetylaspartate; Lac, lactate; Cho, choline; Cr, creatine.

**Figure 4:**
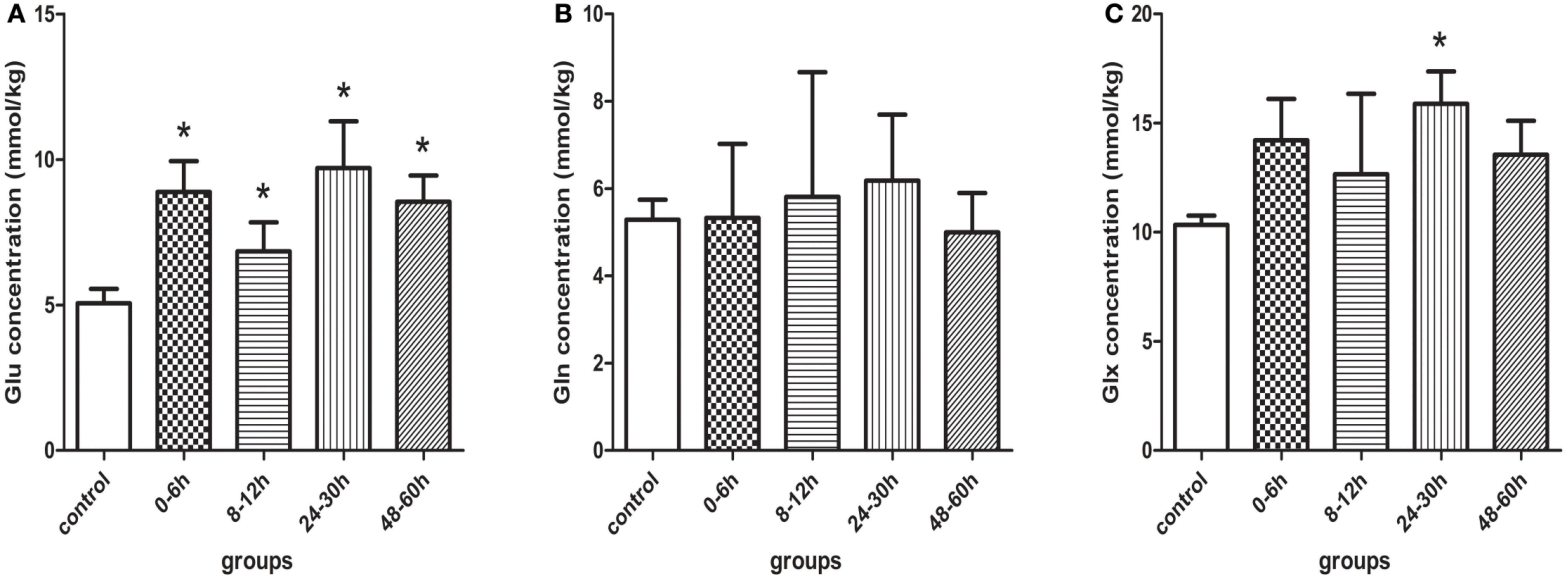
**Quantification results measured by ^1^H-MRS in control and model piglets at different time-points after HI insult**, **(A)** Glu, **(B)** Gln, and **(C)** Glx. Errors bars indicate standard deviation values. ^*^Compared with the control group, *P* < 0.05. Glu, glutamate; Gln, glutamine; Glx, glutamate + glutamine.

The representative ^1^H-MRS in the basal ganglia at different time points after HI are shown in Figure 3. Figure 4 shows the mean levels of Glu, Gln, and Glx in the control group and model group. After HI, there was a sharp increase in Glu level in the basal ganglia at 0–6 h, followed by a transient decrease at 8–12 h, to a level that was still higher than that in the control group; thereafter, it increased again, demonstrating a “two-phase” change. Compared with the control group, the Glu level was increased at different time points after HI, and differences were statistically significant (0–6 h group, *P* < 0.001; 8–12 h group, *P* = 0.016; 24–30 h group, *P* < 0.001; 48–60 h group, *P* < 0.001). There was a statistically significant difference between the HI 0–6 and 8–12 h groups (*P* = 0.007) and between HI 8–12 and 24–30, or 48–60 h groups (*P* < 0.001, *P* = 0.021, respectively), but no statistically significant difference was observed between other groups.

Figure 4B demonstrated that Gln level tended to increase slightly and then decrease after HI, but there were no statistically significant differences between the different groups.

Figure 4C showed that there was a similar change in Glx level as in Glu level, where the difference between the control group and the HI 24–30 h group was statistically significant (*P* = 0.009).

### IVIM data processing

**Figure 5:**
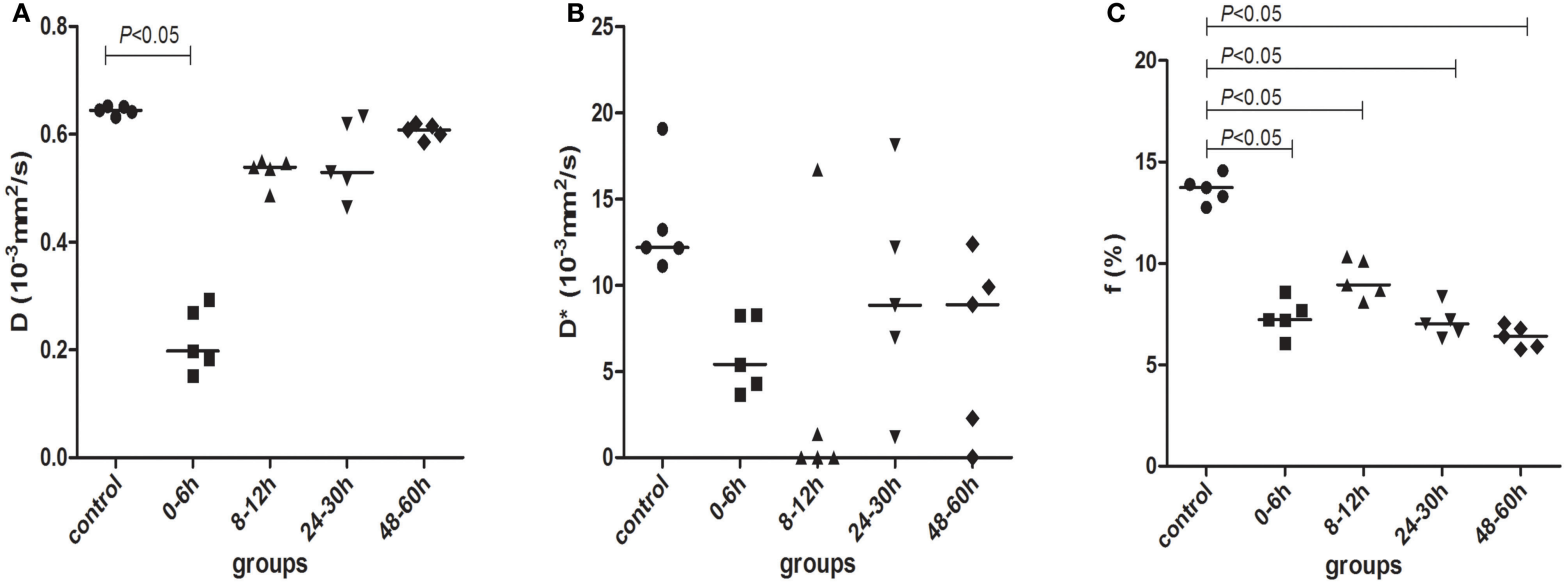
***D*** (A), D^*^ (B), and *f* (C) parameters measured by IVIM scanning in control (black circles) and model piglets at different time-points, 0–6 h (black squares); 8–12 h (black regular triangles); 24–30 h (black inverted triangles); and 48–60 h (black diamonds). The horizontal lines in the scatter plots represent medians.

Figure 5 shows the changes in D, D^*^, and *f* in the control group and model group as detected by IVIM. After HI, *D* in the basal ganglia was markedly decreased at 0–6 h (compared with the control group, *P* < 0.001) and then gradually recovered over time, but it was still slightly lower than that in the control group (no statistically significant differences between different HI time point subgroups and the control group, *P* > 0.05).

Figure 5B showed the changes in D^*^ in the different groups. After HI, D^*^ was decreased at 0–6 and 8–12 h, then increased at 24–30 h and thereafter decreased again at 48–60 h. However, there were no statistical differences in D^*^ between the different groups.

As shown in Figure 5C, *f* was clearly decreased at 0–6 h after HI and then began to recover at 8–12 h, but it was still lower than that in the control group; at 24–30 and 48–60 h, *f* continued to decrease again. The difference in *f* was statistically significant between the control group and the HI model group at different time points (*P* < 0.001, for both). There was a statistically significant difference between 0–6 and 8–12 h groups and between 8–12 and 24–30, or 48–60 h groups (*P* < 0.001, for both). The specific data for D, D^*^, and *f* are shown in Table 1.

**Table 1:** D, D^*^, and *f* parameters measured in newborn piglets from all groups.

**Parameter**		**Model group**			
	**Control group (*n* = 5)**	**0–6 h (*n* = 5)**	**8–12 h (*n* = 5)**	**24–30 h (*n* = 5)**	**48–60 h (*n* = 5)**
*D* (×10^−3^mm^2^/s)	0.644 ± 0.008	0.219 ± 0.060^*^	0.532 ± 0.026^#^	0.552 ± 0.071	0.606 ± 0.014
D^*^ (×10^−3^mm^2^/s)	13.546 ± 3.175	5.979 ± 2.174	3.609 ± 7.336	9.459 ± 6.276	6.683 ± 5.284
*f* (%)	13.650 ± 0.676^#^	7.350 ± 0.914^*^	9.231 ± 0.957^*^^#^	7.103 ± 0.778^*^	6.375 ± 0.551^*^

*Data are shown as mean ± standard deviation*.

*Compared with the control group*,

* P < 0.05

*Compared with HI 48–60 h group*,

# P < 0.05

D, diffusion coefficient; D^*^, pseudo-diffusion coefficient; f, perfusion fraction.

### Analysis of correlations between metabolites of glu metabolism and IVIM-derived perfusion parameters, D^*^ and *f*

**Figure 6:**
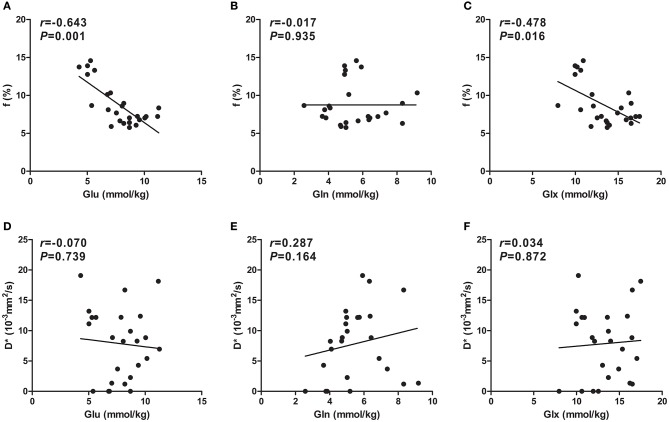
Scatter plots of IVIM-derived *f* (%) and D^*^ (×10^−3^mm^2^/s) parameters over concentrations (mmol/kg) of Glu, Gln, and Glx. A significant negative correlation between *f* values and the Glu concentration was observed **(A)**, whereas an ordinary negative correlation between *f* values and the Glx concentration was observed **(C)**. However, there was no correlation between *f* values and the Gln concentration **(B)**. Unlike for *f* values, no correlation between D^*^ values and Glu **(D)**, Gln **(E)**, or Glx **(F)** concentrations was observed. Spearman's rank correlation coefficient *r* was calculated. Glu, glutamate; Gln, glutamine; Glx, glutamate + glutamine.

After HI, the Glu concentration in the basal ganglia showed a significant negative correlation with *f* (*r* = −0.643, *P* = 0.001; Figure 6A); similarly, there was an ordinary negative correlation between the Glx concentration and *f* (*r* = −0.478, *P* = 0.016; Figure 6C). However, no significant correlation was observed between the Gln concentration and *f* (Figure 6B). In addition, D^*^ did not correlate with the concentration of Glu, Gln, or Glx (Figures 6D–F).

## Discussion

In the present study, we established a HI newborn piglet model and then investigated the level of Glu metabolism and the microcirculatory perfusion changes in the brain by multi-sequence MRI. The results showed that the change in Glu level was closely related to the microcirculatory perfusion level in the acute stage of HIBD. Since the occurrence of HIBD is a result of the interactions and influences of multiple factors that contribute to an abnormal pathological environment in the brain. On one hand, the development of dysmetabolism in cerebral energy causes a massive extracellular accumulation of Glu. The resultant excitotoxicity is one of the key factors inducing neuronal injury and death. On the other hand, hemodynamic disorders of the brain can also lead to damaged brain cells. In the brain, the neurons are located nearby small blood vessels, so the neurotransmitters released by synapses can also regulate the functions of these vessels (Huang et al., 1994) and nerve activities are tightly coupled with the degree of vascular perfusion (Busija et al., 2007; Jackman and Iadecola, 2015). In this study, we performed a preliminary exploration of relevant pathological mechanisms by multi-sequence MRI.

Our study results showed that Glu level underwent a “two-phase” change after HI. The sharp rise in Glu level in the 0–6 h group can be explained by the following mechanism. Energy dysmetabolism in nerve cells occured in the early stage, and the neurons were then depolarized, which caused a substantial release of excitable Glu into the extracellular spaces (Phillis et al., 1994; D'souza and Slater, 1995; Guo et al., 2011; Rocha-Ferreira and Hristova, 2016). Meanwhile, increased NADH/NAD^+^ ratio may directly promote the de novo synthesis of Glu (Ottersen et al., 1996), and the Glu level in astrocytes was then increased accordingly. In the 8–12 h group, the Glu concentration reached a transiently low peak due to the work of Glu transporters (GluTs), responsible for taking up extracellular Glu into astrocytes. On this basis, excessive Glu is transformed into Gln and then returns back to neurons, completing the “Glu (in neuron)–Gln (in astrocyte)” cycle (Bacci et al., 2002). Excessive Glu then suppresses the excessive excitation of basal ganglia, in a possible self-protective mechanism within human astrocytes (Sofroniew and Vinters, 2010; Ouyang et al., 2014). In the 24–30 h group, the Glu concentration increased again due to two causes: cell rupture caused by reperfusion injury led to an increase in Glu release; and the Glu reuptake mechanism was damaged in the late stages of the disease's course and thus resulted in the massive extracellular accumulation of Glu (Matsumoto et al., 1996). Meanwhile, the severe shortage of ATP inhibited the activity of glutamine synthetase (which is responsible for transforming Glu into non-excitatory Gln in the Glu-Gln cycle) (Dao et al., 1991), thus causing the accumulation of residual Glu in astrocytes (Torp et al., 1993).

Moreover, we did not find statistically significant differences in Gln concentration between the different groups in the present study, and this was perhaps associated with the PRESS sequence (*TE* = 37 ms) used for the ^1^H-MRS scan. A PRESS sequence (*TE* = 37 ms) is not the best choice to study Gln. Henry et al. (2011) believed that two-dimensional (2D) J-resolved MRS could completely disperse the peak overlapping on the 2D plane, which is caused by the approximate coupling constants in a one-dimensional plane, and had good stability and no susceptibility to linewidth fluctuation. Therefore, 2D J-resolved MRS is the optimal spectrum study method available for Gln quantification. In the future, we may be able to further investigate and explore Gln by 2D J-resolved MRS.

However, changes in local microcirculatory perfusion in the brain and their association with Glu level have not yet been fully clarified. In this study, we quantitatively evaluated changes in microcirculatory perfusion after HIBD using IVIM. As is well known, water molecules flow with blood (except for Brownian motion) within the capillary network of the microcirculation, and thus capillary blood flow can be regarded as another form of water diffusion. At the voxel level, the water flowing in randomly oriented capillaries can be regarded as an irregular random motion (Le Bihan et al., 1988) known as “pseudo-diffusion” due to the pseudo random organ distribution of the capillary network, and is related to the structure and blood flow rate of the capillary network. In the present study, perfusion was defined as the incoherent motion of water molecules in capillaries at the voxel level (Hu et al., 2015). The analysis of multiple *b*-value diffusion-weighted imaging (DWI) with an IVIM model is applicable to the quantification of two motion components, including water molecule diffusion and microcirculatory perfusion. Three parameters (D, D^*^, and *f*) are then finally calculated using a Levenberg–Marquardt non-linear least square fitting routine, of which D^*^ and *f* both provide information on microcirculatory perfusion. The perfusion fraction *f* represents the volume percentage of perfusion-related diffusion effect in total diffusion effect, and it is positively correlated with the cerebral blood volume of the brain (Federau et al., 2014a,b); the pseudo-diffusion coefficient D^*^, i.e., the capillary perfusion-related diffusion coefficient, is determined depending on the blood flow rate and geometry of capillaries. Therefore, the changes in *f* and D^*^ can be regarded as the changes in microcirculatory perfusion.

This study showed that after HI, *f* decreased at 0–6 h and then transiently recovered at 8–12 h but was still lower than that in the control group, and thereafter, it continued to decrease (see Table 1 and Figure 5C). Several investigators (Pulsinelli et al., 1982; Qiao et al., 2004; Ohshima et al., 2012) have measured changes in local perfusion in the HI brain of animal models using 4-iodo-[14C]-antipyrine, ASL and laser speckle flowmetry (LSF), respectively. They found a transient recovery in cerebral perfusion volume, followed by a decrease in perfusion, which is consistent with our study results. A transient increase in cerebral perfusion volume in the early stages of HIBD may be attributed to the activation of a cerebral vascular self-regulatory mechanism (Grant et al., 2005) (a self-protective mechanism of the body) after the recovery of blood reperfusion in the bilateral common carotid arteries. Blood perfusion decreased in the late stages, after HI. Such delayed hypoperfusion in the clinic is controversial, but most evidence suggests that it is closely related to secondary brain damage after HI (Jensen et al., 2006).

In the present study, the Glu concentration in the basal ganglia of newborn piglets significantly correlated with *f* in a negative manner (*r* = −0.643, *P* = 0.001). At 8–12 h after HI, *f* increased transiently (Table 1 and Figure 5C), corresponding to a transient low peak of Glu release (see Figure 4A), which indicates that the excessively accumulated Glu was cleared due to the recovery of perfusion in the early stages after HI. (Yamaguchi, 1977) The majority of current studies (Busija et al., 2007; Longo and Goyal, 2013) have shown that Glu and its synthetic analogs can dilate small arteries and veins in the brain and thus increase *f*. The Glu concentration increased while *f* decreased again, perhaps because delayed hypoperfusion can mediate the release of this excitatory neurotransmitter (Matsumoto et al., 1996). In turn, the abovementioned vasodilatory effect of Glu may be weakened with the progression of disease, and Glu may even constrict small arteries in the cerebral pia mater. As the Glu concentration increased, cerebral vasospasms may have become aggravated and may have even induced the breakage of postcapillary venules, thus causing severe microcirculatory disturbances (Huang et al., 1994), decreasing *f* further. Based on this finding, we postulate that the disturbances in microcirculatory perfusion and Glu release may result from, and contribute to, each other, and both may induce neuronal injury following HI.

However, our study results showed no statistically significant difference in D^*^. A possible reason may be the big limitation of D^*^, which results from uncertainty and very poor reproducibility (Wu et al., 2015; Nougaret et al., 2016; Yang et al., 2016). We found that D^*^ displayed a large degree of dispersion during post-processing computations, maybe because of a high variation in D^*^ within brain tissues due to the blood–brain barrier; *f* usually shows a small value in brain tissues. That is to say, D^*^ is more likely to have an error when the proportion of perfusion is lower (King et al., 1992; Bisdas et al., 2013). In addition, the movements of animals and the partial volume effect of CSF during scanning can influence the measuring accuracy of D^*^. Some investigators have proposed that the accurate measurement of D^*^ was also affected by the cardiac cycle, with the measurement value of D^*^ significantly higher in the systole than in the diastole (Federau et al., 2013; Xu et al., 2016). Relative to D^*^, *f* was less affected by these physiological factors, had lower noise and was more uniform.

In addition, this study also demonstrated that the diffusion coefficient *D*, a parameter reflecting the true diffusion motion of water molecules, was markedly decreased at 0–6 h after HI and then gradually recovered over time, but was still slightly lower than that in the control group (see Figure 5A). This was perhaps due to increased glycolysis in the tissues in the early stage of HI, with more and more lactate accumulated, resulting in intracellular acidosis and cytotoxic edema. This subsequently led to the reduction of extracellular spaces (Tuor et al., 2014), which limited the diffusion of water molecules and decreased *D*. Subsequently, cellular swelling caused by cytotoxic edema compressed the capillaries and then resulted in the further hypoxia of brain tissues. Such hypoxia acted on vascular endothelial cells to increase vascular permeability, resulting in vascular edema. As a result, water molecules were retained extracellularly, the extracellular space became enlarged (Wang H. et al., 2012), the diffusion of water molecules was enhanced, and *D* increased. Conventional DWI reflects the microstructural changes in brain tissues with changes of the apparent diffusion coefficient (ADC). However, diffusions in biotic tissues actually measured by ADC include both the true diffusion of water molecules and capillary perfusion effects, which can be distinguished by IVIM (Le Bihan et al., 1986, 1988). Therefore, multiple *b*-value DWI, based on an IVIM bi-exponential model, can provide a value of *D* without the effects of perfusion factors, and thus can more accurately measure the diffusion of water molecules. In short, the early pathological changes in brain tissues after HI are indicated by changes in *D*.

Of course, our study had several limitations. Firstly, a small sample size was used, which may have caused a bias in results. However, we hope our results lay the basis for further, larger studies. Secondly, the coil diameter used in this study was relatively bigger than the head of the newborn piglets, and the SNR of images was not high enough. Therefore, work must focus on seeking a better SNR for future studies. Finally, the IVIM sequence was scanned with 16 *b*-values (including 9 *b*-values within a range of 0–200 s/mm^2^) in the present study, which took a long time, though more accurate and detailed perfusion information was provided; the motion displacement during scanning may have affected the accuracy of results. However, in order to overcome this limitation, data with severe motion was excluded.

## Conclusion

In this study, we evaluated the changes in Glu metabolism and microcirculatory perfusion in the HI brain by ^1^H-MRS combined with IVIM. Glu concentration was increased in the early stage after HI, then transiently recovered and finally increased again, showing a “two-phase” change; perfusion-related parameter *f* showed a clear decrease, then transiently and slightly recovered, and thereafter continued to decrease. There was a significant negative correlation between the two parameters. Our data highlight the potential of combining changes in Glu concentration and *f* to explore the close relationship between cerebral dysmetabolism and microcirculatory disturbance after HI.

## Author contributions

XW and YD participated in conceiving and designing of the idea. KS contributed to providing the post-processing assistant. YD and KS contributed to analyzing the experiment results. YD also contributed to drafting and editing of the manuscript. XW and YD also participated in critically revising the paper. All authors have read and approved the final manuscript for publication.

## Funding

The author(s) disclosed receipt of the following financial support for the research, authorship, and/or publication of this article: This work was supported by the National Natural Science Foundation of China (grant no. 30570541, 30770632, 81271631) and Outstanding Scientific Fund of Shengjing Hospital (item no. 201402).

### Conflict of interest statement

The authors declare that the research was conducted in the absence of any commercial or financial relationships that could be construed as a potential conflict of interest.
